# A State University Perspective on Becoming an Age-Friendly University

**DOI:** 10.1093/geroni/igaa057.1790

**Published:** 2020-12-16

**Authors:** Neil Charness

**Affiliations:** Florida State University, Tallahassee, Florida, United States

## Abstract

I describe the process of FSU becoming an Age-Friendly University (AFU). After hearing a presentation on AFU at the Age Directors meeting at GSA by Joann Montepare in November 2016, I judged that many of the ten principles of an AFU were already in operation at FSU. So, I decided to confer in December with VP for Faculty Development and Advancement, Janet Kistner, about how to move the AFU application process forward. I generated a draft document and circulated it to major units dealing with aging on campus: the Institute for Successful Longevity, the Pepper Institute on Aging and Public Policy, and the Pepper Center. Eventually, a one-page version of the longer document was introduced at an FSU Cabinet meeting and approved in April 2017. I detail some of the critical discussion points, the concerns of upper administration, and the timeline for completion of the process at FSU. Part of a symposium sponsored by Directors of Aging Centers Interest Group.

